# Effects of Basil (*Ocimum basilicum* L.) Leaf Extracts on Gastrointestinal Smooth Muscle Spasms: An In Vitro Study on Rat Ileum

**DOI:** 10.3390/plants15071079

**Published:** 2026-04-01

**Authors:** Milica Randjelović, Nebojša Simić, Suzana Branković, Maja Koraćević, Miloš Jovanović, Nemanja Kitić, Bojana Miladinović, Milica Milutinović, Dušanka Kitić

**Affiliations:** 1 Department of Pharmacy, Faculty of Medicine, University of Nis, Ave. Dr. Zorana Djindjica 81, 18000 Nis, Serbia; milos.jovanovic@medfak.ni.ac.rs (M.J.); bojana.miladinovic@medfak.ni.ac.rs (B.M.); milica.milutinovic@medfak.ni.ac.rs (M.M.); dusanka.kitic@medfak.ni.ac.rs (D.K.); 2 Department of Chemistry, Norwegian University of Science and Technology, 7491 Trondheim, Norway; 3 Department of Physiology, Faculty of Medicine, University of Nis, Ave. Dr. Zorana Djindjica 81, 18000 Nis, Serbia; brankovic.suzana@yahoo.com (S.B.); nemanja.kitic@medfak.ni.ac.rs (N.K.); 4 Faculty of Medicine, University of Nis, Ave. Dr. Zorana Djindjica 81, 18000 Nis, Serbia; koracevic.maja@gmail.com

**Keywords:** *Ocimum basilicum* L., leaf extracts, spasmolytic activity, ileum, rats

## Abstract

The present study was designed to evaluate the effects of eighteen different extracts derived from basil (*Ocimum basilicum* L.) leaves on spontaneous contractions, as well as contractions induced by potassium chloride (KCl) and acetylcholine in the ileum of rats, under in vitro conditions. The extracts were prepared with 96% *v*/*v*, 80% *v*/*v*, and 60% *v*/*v* ethanol, and absolute (100%) *v*/*v*, 80% *v*/*v*, and 60% *v*/*v* methanol, employing extraction techniques that included maceration, digestion, and sonication-assisted methods. Chemical characterization of the extracts revealed the presence of various phenolic acids, including rosmarinic, chlorogenic, caftaric, salvianolic acid B, cinnamic, caffeic, and chicoric acid, as well as flavonoids such as rutin and salvigenin. The evaluated extracts produced significant, concentration-dependent inhibitory effects on rat ileal contractions. Notably, the extract obtained via maceration with 80% methanol exhibited the most pronounced relaxant effects on spontaneous muscle contractions, achieving a maximum reduction of 46.16 ± 2.11%. Furthermore, the extract prepared with the same solvent using sonication-assisted extraction demonstrated superior efficacy in diminishing both the frequency and amplitude of KCl-induced ileal contractions, reducing contraction intensity caused by elevated potassium ion levels to 59.48 ± 3.34% at a maximum concentration of 1.5 mg/mL, thereby indicating its potential as a potent calcium channel blocker. Additionally, the extract prepared with 60% methanol through sonication-assisted extraction resulted in the most substantial reduction of acetylcholine-induced ileal contractions, decreasing contraction intensity to 35.74 ± 1.54% at the maximum concentration of 1.5 mg/mL, which suggests a high level of neurophysiological activity. By comparing extracts with different phytochemical profiles, this study provides additional insight into how variations in phenolic composition may influence different mechanisms of smooth muscle relaxation. This study affirms the significant spasmolytic properties of basil leaf extracts, thereby supporting their potential application in the management of gastrointestinal motility disorders.

## 1. Introduction

The genus *Ocimum* L. of the family Lamiaceae comprises more than 30 species of herbs and shrubs native to Asia, Africa, and Central and South America [1]. *Ocimum basilicum* L., commonly known as basil, is an aromatic annual plant widely used as a spice and considered a typical ingredient of the health-promoting Mediterranean diet [2]. In traditional medicine in Serbia, preparations of the aerial parts of basil have been used internally for menstrual and digestive disorders accompanied by stomach pain, for chills and anxiety, and as an inhalation agent in the treatment of productive cough, pharyngitis, bronchitis, and sinusitis [3,4]. Previous pharmacological studies indicate that basil products show potential for neuroprotective, cardioprotective, and antidiabetic activities, beneficial actions on the respiratory and gastrointestinal systems, as well as anti-inflammatory, antinociceptive, antimicrobial, antitumor, chemopreventive, antioxidant, and dermatological properties [2].

The most commonly used parts of basil in pharmacy are leaves, providing essential oils and phenolic compounds, and seeds serving as a source of mucilage [1,5]. Basil essential oil is rich in terpenic compounds, including monoterpenes and sesquiterpenes such as linalool, geraniol, limonene, caryophyllene, and *α*-cadinol. Although widely used, its application may be limited due to the potential toxicity of alkenylbenzenes, such as eugenol and estragole, which it may contain [2,5]. Conversely, the consumption of basil in foods and its hydroalcoholic extracts, owing to the protective effect of the phenolic plant matrix, does not pose a risk to human health and, instead, can provide multiple health benefits [2]. The health benefits of basil are primarily associated with its content of flavonoids and phenolic acids. The most abundant flavonoids include quercetin, rutin, catechin, kaempferol, and luteolin, whereas the principal phenolic acids are rosmarinic acid and other caffeic acid derivatives [5]. The profile and content of secondary metabolites in basil are strongly influenced by chemotypic variation [6].

Proper intestinal motility is pivotal for gastrointestinal physiological processes, including digestion, nutrient absorption, and excretion. Disorders of intestinal motility are frequently associated with conditions such as irritable bowel syndrome, inflammatory bowel disease, and constipation. Existing treatment strategies, encompassing both pharmacological and surgical options, are limited by suboptimal efficacy and potential adverse effects [7]. Numerous medicinal plants have long been empirically used as spasmolytic agents in managing smooth muscle spasm–related disorders. Experimental validation of their pharmacological activity, along with the identification of molecular targets and mechanisms of action of active compounds, rationalizes their traditional use and may guide the future development of more effective and safer drugs [8].

Numerous studies have investigated the therapeutic potential of basil in gut-related disorders associated with prebiotic effects modulating gut microbiota composition [9], while its impact on gastrointestinal motility remains poorly explored [5,10]. Therefore, the aim of the present study was to evaluate the effects of basil leaf extracts, prepared with different methods and solvents, on gastrointestinal contractions and to investigate the principal mechanisms of their action. The polyphenol profile of the alcoholic and hydroalcoholic extracts, with phenolic acids and flavonoids as dominant constituents, was analyzed to evaluate their potential contribution to the observed spasmolytic activity. Furthermore, the objective was to examine the impact of solvent type and extraction method on the content of identified pharmacologically active constituents.

## 2. Results

### 2.1. Chemical Characterization of the O. basilicum Leaf Extracts

The results of the chemical analysis and extraction yields of basil leaf extracts obtained by ultrasound-assisted extraction (U), digestion (D), and maceration (M) using different concentrations of ethanol (E; 96%, 80%, and 60% *v*/*v*) and methanol (M; absolute, 80%, and 60% *v*/*v*) are presented in Table 1. Extracts are labeled according to the solvent and its concentration, followed by the extraction technique: EU, E80U, E60U; ED, E80D, E60D; EM, E80M, E60M for ethanol extracts, and MU, M80U, M60U; MD, M80D, M60D; MM, M80M, M60M for methanol extracts. As expected, both the extraction method and solvent markedly affected extraction yields, which varied more than twelvefold, ranging from 1.16% *w*/*w* (EU) to 14.43% *w*/*w* (M80M). In general, extraction yields followed the trend of maceration > digestion > ultrasound-assisted extraction for all solvents, except for 60% *v*/*v* ethanol, where digestion > ultrasound-assisted extraction > maceration was observed. Methanolic extracts showed higher yields than their ethanolic counterparts at the same concentration and extraction method, except in the case of digestion with 60% *v*/*v* solvent.

**Figure 1:**
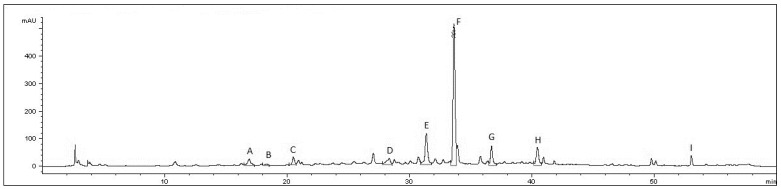
Representative HPLC chromatogram of an *Ocimum basilicum* L. extract (M80M—extract prepared with 80% *v*/*v* methanol by maceration), recorded at 280 nm. A. caftaric acid, B. chlorogenic acid, C. caffeic acid, D. rutin, E. chicoric acid, F. rosmarinic acid, G. salvianolic acid B, H. cinnamic acid and I. salvigenin.

The results indicate that basil leaf is a rich source of phenolic acids (chlorogenic, caffeic, rosmarinic, salvianolic acid B, cinnamic, caftaric, and chicoric acids) and flavonoids (salvigenin and rutin). HPLC-DAD analysis showed no significant qualitative differences among the analyzed samples, while quantitative discrepancies were evident (Figure 1). Rosmarinic acid was identified as the dominant secondary metabolite in all samples, with concentrations ranging from 17.02 ± 0.08 µg/mg (EU) to 77.75 ± 0.91 µg/mg (E60M). With the exception of extracts obtained using 96% *v*/*v* ethanol, rosmarinic acid was consistently followed by salvianolic acid B as the second most abundant phenolic acid in all other extracts, with concentrations ranging from 0.91 ± 0.05 µg/mg to 9.94 ± 0.10 µg/mg. In terms of flavonoid content, rutin was the dominant compound in all samples (1.27 ± 0.06 to 5.66 ± 0.05 µg/mg), except for the EU sample, in which salvigenin predominated. Regarding the sum of all monitored compounds, the highest concentration (110.38 µg/mg) was observed in E60M, whereas the highest extraction efficiency (expressed as the concentration of compounds adjusted for extract yield) was achieved with M80M.

**Figure 2:**
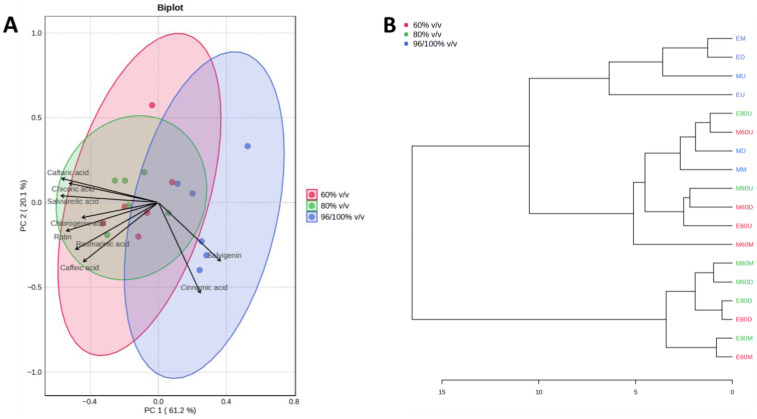
PCA biplot of the *Ocimum basilicum* L. leaf extracts showing sample grouping according to extraction methods (**A**). The corresponding hierarchical clustering dendrogram based on Euclidean distance and Ward’s method is shown in (**B**), where the horizontal axis represents Euclidean distance, with lower values indicating higher similarity and higher values indicating greater dissimilarity between samples; (EU, ED, EM—extracts prepared with 96% *v*/*v* ethanol by ultrasound (sonication)-assisted extraction, digestion and maceration, respectively; E80U, E80D, E80M—extracts prepared with 80% *v*/*v* ethanol by ultrasound-assisted extraction, digestion and maceration, respectively; E60U, E60D, E60M—extracts prepared with 60% *v*/*v* ethanol by ultrasound-assisted extraction, digestion and maceration, respectively; MU, MD, MM—extracts prepared with absolute methanol by ultrasound-assisted extraction, digestion, and maceration, respectively; M80U, M80D, M80M—extracts prepared with 80% *v*/*v* methanol by ultrasound-assisted extraction, digestion and maceration, respectively; M60U, M60D, M60M—extract prepared with 60% *v*/*v* methanol by ultrasound-assisted extraction, digestion, and maceration, respectively).

**Figure 3:**
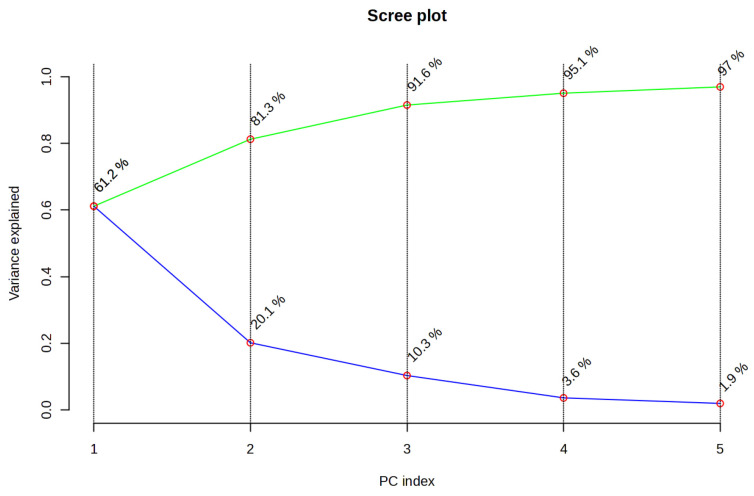
Scree plot of the first five principal components (PC1–PC5) for the *Ocimum basilicum* L. leaf extracts, showing individual variance (blue line) and cumulative variance (green line).

**Figure 4:**
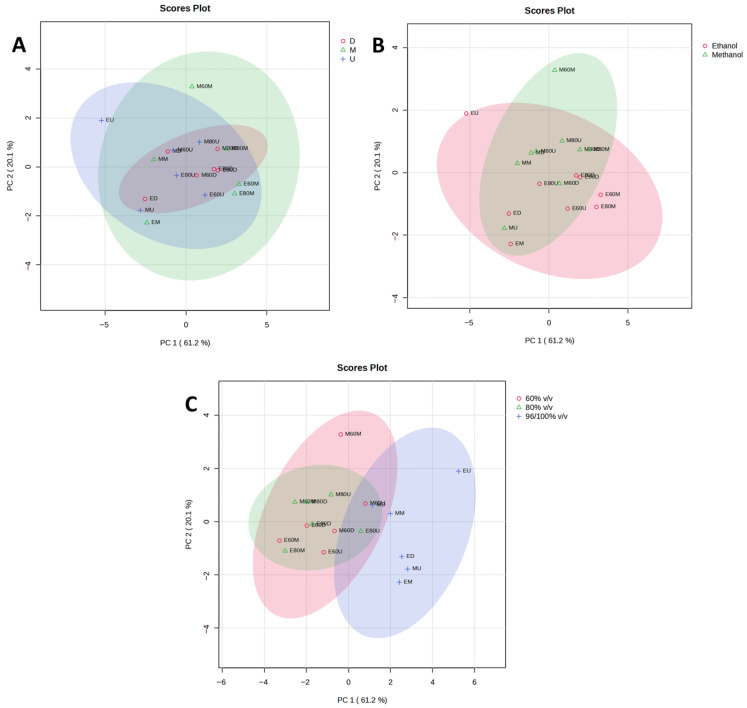
PCA score scatter plots of the *Ocimum basilicum* L. leaf extracts, showing sample grouping by extraction methods (**A**), solvent type (**B**), and solvent concentration (**C**).

**Figure 5:**
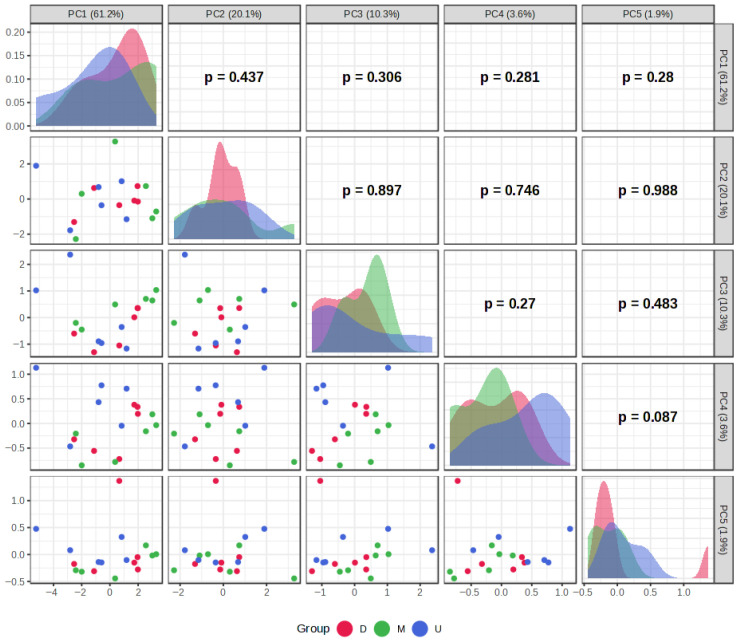
Pairwise visualization of PCA scores (PC1–PC5) of samples grouped by extraction method, illustrating the distribution and relationships of scores across the principal components.

**Figure 6:**
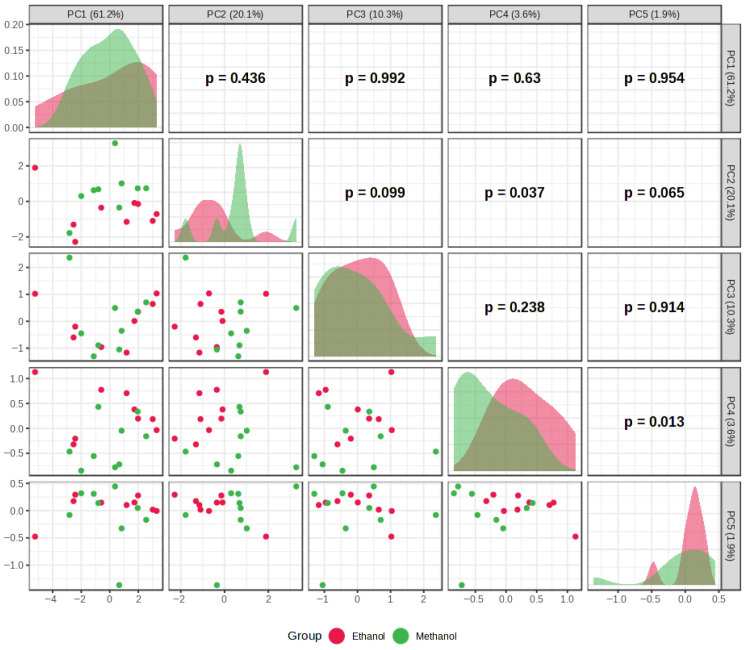
Pairwise visualization of PCA scores (PC1–PC5) of samples grouped by solvent type, illustrating the distribution and relationships of scores across the principal components.

**Figure 7:**
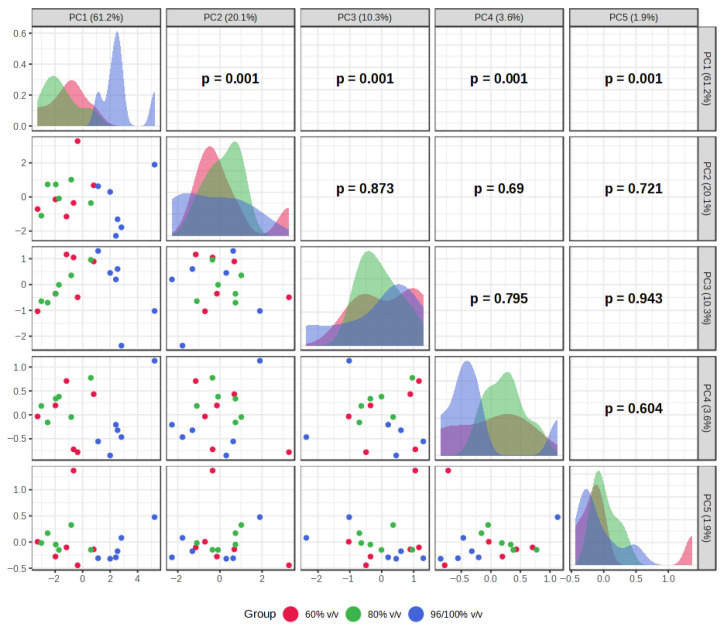
Pairwise visualization of PCA scores (PC1–PC5) of samples grouped by solvent concentration, illustrating the distribution and relationships of scores across the principal components.

The results of PCA and HCA, used to identify patterns in extraction efficiency under different extraction conditions, are presented in Figure 2, Figure 3, Figure 4, Figure 5, Figure 6 and Figure 7. PCA reduced the dataset to two principal components explaining 81.3% of the total variance, with PC1 and PC2 accounting for 61.2% and 20.1%, respectively (Figure 2A), while the first five principal components (PC1–PC5) together explained 97% of the total variance (Figure 3). Clear clustering of samples according to solvent concentration (Figure 4C), contrasted with the overlap observed for extraction method and solvent type (Figure 4A,B), indicates that solvent concentration is the main factor influencing the variability in the chemical profiles of the analyzed extracts. According to the PCA biplot (Figure 2A), salvigenin and cinnamic acid had strong positive loadings on PC1, whereas caftaric and salvianolic acid B loaded negatively, showing opposite contributions. PC2 was primarily driven by caftaric acid (positive) and cinnamic acid (negative). The HCA dendrogram (Figure 2B) revealed distinct clustering patterns among the extracts and further supported the PCA results. Extracts obtained with the highest solvent concentrations (96% ethanol and absolute methanol) formed a clearly separated cluster (EM, ED, MU, EU), indicating that these extraction conditions produced the most distinct chemical profiles. In contrast, extracts prepared with aqueous solvents (60% and 80%) grouped into separate subclusters primarily according to solvent concentration rather than extraction technique. For example, samples obtained with 60% solvents (E60 and M60 series) clustered together, while those prepared with 80% solvents formed closely related groups regardless of whether ultrasound-assisted extraction, digestion, or maceration was applied. The distribution of samples within these clusters indicates that solvent concentration had a stronger influence on the phytochemical composition of the extracts than the extraction method itself, which is consistent with the trends observed in the PCA analysis.

### 2.2. Effects of the O. basilicum Leaf Extracts on Spontaneous Rat Ileal Contractions

**Figure 8:**
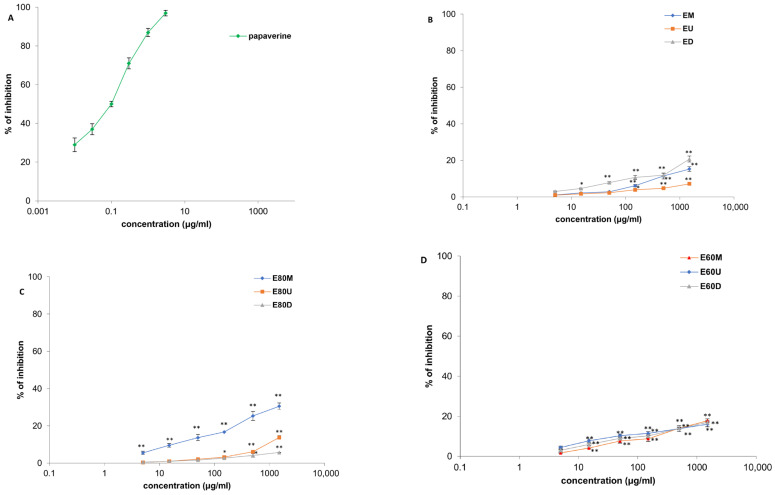
Relaxant effects of papaverine (**A**), *Ocimum basilicum* L. extracts, EU, ED, EM (**B**), E80U, E80D, E80M (**C**), and E60U, E60D, E60M (**D**) on spontaneous contractions of rat ileum. Each point denotes the mean percentage of spontaneous contractions in Tyrode solution (control) ± SD obtained from six segments; differences were assessed by Student’s *t*-test (* *p* < 0.05, ** *p* < 0.01 vs. Tyrode).

**Figure 9:**
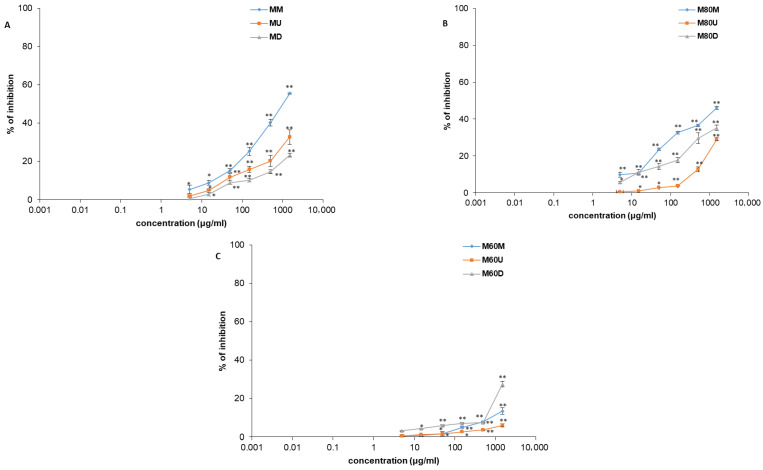
Relaxant effects of *Ocimum basilicum* L. extracts MU, MD, MM (**A**), M80U, M80D, M80M (**B**), and M60U, M60D, M60M (**C**) on spontaneous contractions of rat ileum. Each point denotes the mean percentage of spontaneous contractions in Tyrode solution (control) ± SD obtained from six segments; differences were assessed by Student’s *t*-test (* *p* < 0.05, ** *p* < 0.01 vs. Tyrode).

Investigated extracts exhibited the significant, dose-dependent spasmolytic effects on ileal smooth muscle in the evaluation of their effects on spontaneous contractions (0.005–1.5 mg/mL) (Figure 8 and Figure 9, Table 2). Although the spasmolytic effects of the extracts were significant, they were less potent compared to the nonspecific myorelaxant papaverine. Papaverine, at a maximum applied concentration of 0.003 mg/mL, inhibited 97.01 ± 0.98% of contractions. Among the tested extracts, M80M, prepared by maceration using 80% *v*/*v* methanol, showed the most pronounced effect with a maximal inhibition of 46.16 ± 2.11% at a concentration of 1.5 mg/mL. When considering the effects of ethanolic extracts on spontaneous contractions of isolated rat ileum, the E80M extract, prepared by the same extraction method using 80% *v*/*v* ethanol, exhibited the highest efficacy. This extract produced a 30.55 ± 1.69% reduction in ileal contractions at a maximal concentration of 1.5 mg/mL.

### 2.3. Effects of the O. basilicum Leaf Extracts on KCl-Induced Rat Ileal Contractions

**Figure 10:**
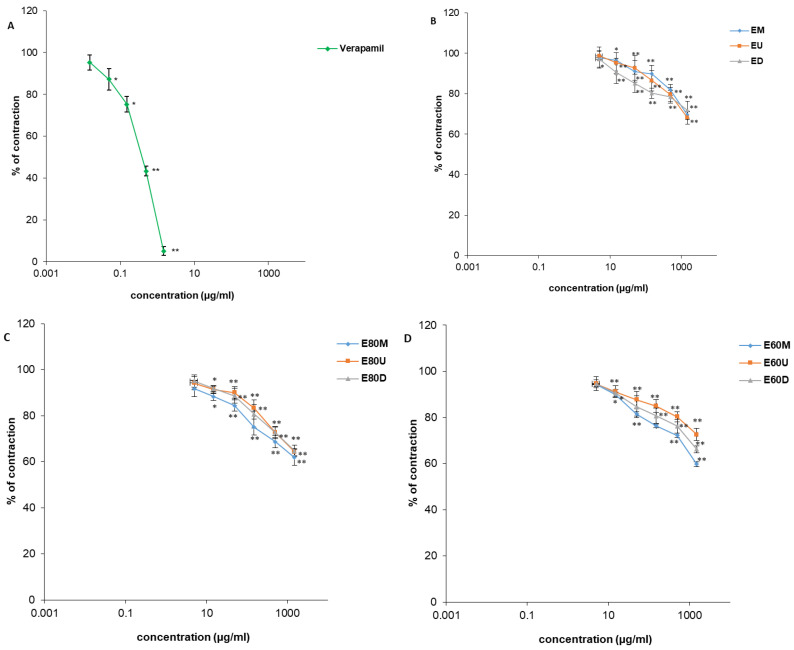
Relaxant effects of verapamil (**A**), *Ocimum basilicum* L. extracts EU, ED, EM (**B**), E80U, E80D, E80M (**C**), and E60U, E60D, E60M (**D**) on KCl-induced contractions of rat ileum. Each point denotes the mean percentage of the maximal response ± SD obtained from six segments; differences were assessed by Student’s *t*-test (* *p* < 0.05, ** *p* < 0.01 vs. control).

**Figure 11:**
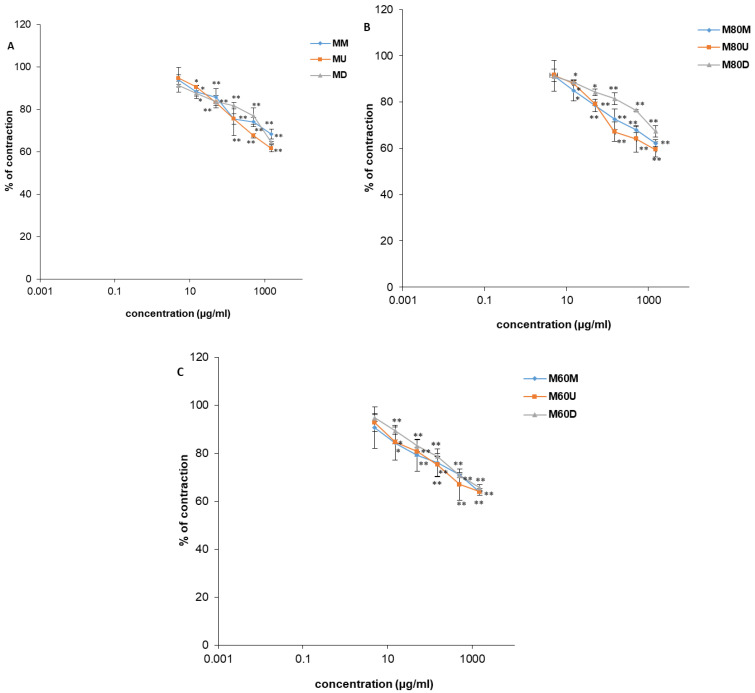
Relaxant effects of *Ocimum basilicum* L. extracts MU, MD, MM (**A**), M80U, M80D, M80M (**B**), and M60U, M60D, M60M (**C**) on KCl-induced contractions of rat ileum. Each point denotes the mean percentage of the maximal response ± SD obtained from six segments; differences were assessed by Student’s *t*-test (* *p* < 0.05, ** *p* < 0.01 vs. control).

As with spontaneous contractions, the *O. basilicum* extracts (0.005–1.5 mg/mL) also exhibited an inhibitory effect on contractions induced by the application of KCl solution (Figure 10 and Figure 11; Table 3). Relaxation of the ileal smooth muscle was dose-dependent, with maximal reduction to levels within a narrow range from 59.48 ± 3.34% to 72.56 ± 2.62%. Verapamil was used as a control in this experimental series and at a maximal concentration of 0.0015 mg/mL, reduced the contraction to 5.04 ± 0.09%. The extract prepared with 80% *v*/*v* methanol using sonication-assisted extraction, M80U, demonstrated superior efficacy in reducing both the frequency and amplitude of induced ileal contractions, reducing contraction intensity to 59.48 ± 3.34% at a maximum concentration (1.5 mg/mL). In addition, among the ethanolic extracts, E60M, obtained using 60% *v*/*v* ethanol by maceration, was distinguished by similar spasmolytic effects.

### 2.4. Effects of the O. basilicum Leaf Extracts on Acetylcholine-Induced Rat Ileal Contractions

The extracts significantly reduced ileal contractions induced by cumulative doses of acetylcholine (5–1500 nM) (Table 4; Figure S1). The addition of the extracts at concentrations of 0.5 and 1.5 mg/mL reduced contractions induced by each tested concentration of acetylcholine. The most pronounced effect was observed with extracts prepared using the sonication-assisted method and 60% *v*/*v* methanol, M60U. This extract produced the strongest reduction of the ileal contraction caused by the maximum acetylcholine dose of 1500 nM (100%) to 68.83 ± 4.98% at the applied dose of 0.5 mg/mL, and to 35.74 ± 1.15% at the applied dose of 1.5 mg/mL. It is also important to emphasize the M80M extract, which at a dose of 0.5 mg/mL more effectively reduced the maximal acetylcholine response, from 100% to 50.71 ± 1.36%. However, at a higher dose, its inhibitory effect was lower compared to that of M60U. Atropine, used as a positive control, reduced the maximum contraction of the ileum to 16.02% with the applied concentration of 140 nM.

## 3. Discussion

Earlier studies [11,12] have shown that extracts rich in phenolic acids and their derivatives, with rosmarinic acid as the major compound, were obtained from basil aerial parts, which is consistent with our results for extracts prepared from basil leaves. In contrast, another study reported that flavonoids predominate among phenolic compounds in ethanolic leaf extracts of basil, with rutin and epicatechin identified as the major constituents [13]. The observed differences in the chemical composition of basil extracts, at both qualitative and quantitative levels, may be attributed to multiple factors, including geographical origin, chemotype, and extraction parameters, which are known to shape the basil phenolic profile [5,6].

The influence of solvent concentration, which PCA indicated as the factor contributing most to the variability in the chemical profiles among the tested extraction conditions, can be attributed to changes in solvent polarity, a key factor affecting the solubility of compounds. Besides solubility, the ability of the solvent to disrupt hydrogen and hydrophobic bonds influences extraction, as polyphenols in the plant matrix are often bound to proteins and/or polysaccharides through such interactions. This explains why binary solvent systems, combining solvents of different polarity and hydrogen-bonding ability, are generally more effective for polyphenol extraction than single-solvent systems [14]. Additionally, ethanol concentrations near 100% *v*/*v* can cause dehydration of the plant tissue and denaturation of proteins, leading to a matrix entrapment effect, resulting in decreased polyphenol extraction efficiency [15].

Considering both the extraction yields of the monitored bioactive compounds and the compliance with green chemistry principles, maceration using 60 or 80% *v*/*v* ethanol appears to be well suited for the extraction of basil leaves. Although slightly higher yields were obtained with 80% *v*/*v* methanol, ethanol represents a more acceptable solvent due to its lower toxicity and generally recognized as safe (GRAS) status [15]. Moreover, the present results indicate that maceration, despite being a conventional technique, remains competitive in terms of extraction efficiency and economic feasibility. While modern extraction methods, such as ultrasound- or microwave-assisted extraction, are often favored because of reduced processing times, these techniques may involve localized thermal or mechanical effects that can adversely affect heat-sensitive phytochemicals, such as many phenolics. In contrast, maceration is conducted under mild conditions, which supports the preservation of compound stability, offers straightforward scalability, and reduces the risk of chemical degradation, making it a suitable option for sustainable and reproducible extraction processes [6,16].

The investigated *O. basilicum* leaf extracts exhibited significant spasmolytic effects, which correlate with their traditional use in the treatment of various digestive disorders [9]. The results indicate that the spasmolytic activity of the extracts largely depends on their chemical composition, particularly on the presence and relative proportions of individual phenolic compounds. The differences observed in the intensity of relaxation of rat ileum smooth muscle in the models of spontaneous, KCl-induced, and acetylcholine-induced contractions suggest that different components of the extracts may influence different regulatory mechanisms of smooth muscle contractility. Intestinal motility is one of the main factors that affects the functioning of the digestive system, as well as proper digestion and absorption of food components. The coordination of smooth muscle contractions is regulated by complex mechanisms that involve numerous receptors for various excitatory or inhibitory substances [17]. Contraction of the isolated intestinal preparation is initiated by an increase of the free calcium (Ca^2+^) levels in cytoplasm which subsequently activates the contractile apparatus. The rise in intracellular calcium levels is mediated either by influx through voltage-dependent L-type calcium channels or by release from intracellular calcium stores within the sarcoplasmic reticulum. Spontaneous intestinal motility is governed by periodic depolarization, during which an action potential is generated at the peak of depolarization as a consequence of rapid calcium entry through these channels [18,19]. In the model of spontaneous ileum contractions, the most pronounced inhibitory effect was observed for extracts rich in rosmarinic acid, M80M. This extract showed the highest extraction efficiency and contained relatively high levels of rosmarinic acid together with considerable amounts of salvianolic acid B and chicoric acid, suggesting that the combined presence of several phenolic acids may enhance the overall spasmolytic effect. Although E60M contained the highest concentration of rosmarinic acid, its spasmolytic activity was not the most pronounced among the tested extracts. This observation indicates that the biological activity of *O. basilicum* leaves extracts cannot be attributed solely to rosmarinic acid, but rather to the combined contribution of several phenolic compounds and their possible synergistic interactions. The presence of other phenolic constituents and their mutual interactions likely play an important role in determining the overall activity of the extracts.

Further insight into the mechanism underlying the spasmolytic activity of *O. basilicum* extracts was obtained from experiments involving KCl-induced contractions, which provide information about their effects on calcium channels. In the present study, all investigated extracts attenuated KCl-induced ileal contractions to varying degrees, indicating that calcium channel inhibition may represent one of the principal mechanisms underlying their spasmolytic activity. Among the tested samples, the M80U extract demonstrated the strongest inhibitory effect. This extract contained considerable amounts of several phenolic acids, including rosmarinic acid, salvianolic acid B, caftaric acid, and chicoric acid, suggesting that these compounds may contribute collectively to the observed calcium-antagonistic activity. High concentrations of potassium ions cause smooth muscle contractions as a result of membrane depolarization, opening of voltage-dependent L-type calcium channels, and Ca^2+^ influx into the cell [20]. This spasmolytic mechanism constitutes the predominant way in which plant extracts and essential oils act on smooth muscle contractions [21,22,23]. Comparable findings were reported by Janbaz et al. [10], who demonstrated relaxant effects of an aqueous–methanolic extract of basil, prepared from aerial parts, on spontaneous and K^+^-induced contractions of rabbit jejunum.

The third series of experiments involved the assessment of the potential neurophysiological activity of the basil extracts. The effects of the extracts on the cholinergic system in the rat small intestine were evaluated in the acetylcholine-induced contractions model. Inhibitory effects on this type of ileal contraction were observed for all tested extracts, suggesting muscarinic receptor blockade. The M60U and M80M extracts exhibited the strongest inhibitory effects in this model. These extracts did not possess the highest sum of all compounds, indicating that the observed activity, as mentioned, may depend not only on the concentration of individual compounds but also on their relative proportions and possible synergistic interactions. Similar to our investigation, aqueous extracts of basil leaf and stem were able to diminish metacholine-induced rats jejunum contractions [5]. In contrast, these findings differ from those of Janbaz et al. [10], who reported a contractile potentiation in isolated guinea pig ileum.

Based on these findings, it can be concluded that the spasmolytic activity of *O. basilicum* extracts arises from the complex and likely synergistic action of several phenolic compounds, with different constituents targeting different mechanisms involved in the regulation of smooth muscle contractility. At the same time, the results clearly demonstrate that extraction conditions significantly influence the chemical composition of the extracts and consequently their pharmacological potential. Extracts obtained with hydroalcoholic solvents of intermediate polarity showed the most favorable relationship between chemical composition and spasmolytic activity, highlighting the importance of optimizing extraction conditions in studies investigating the biological activity of plant extracts.

Identified phytochemicals appear responsible for the spasmolytic effects of the basil extracts. Rosmarinic acid notably suppressed spontaneous, KCl-, and acetylcholine-induced contractions in isolated rat ileum, demonstrating its efficacy against gastrointestinal spasms [24]. Comparable effects were observed in jejunum preparations from swine and ileum preparations from rabbits [25,26]. Chlorogenic acid has also been shown to be an effective spasmolytic agent, as it reduced spontaneous and acetylcholine-induced contractions of swine jejunum [26]. Caffeic acid also suppressed KCl-induced contractions and expressed neurophysiological activity as it reduced the spasmogenic effects of carbachol [27]. Rutin has been shown in several studies to exhibit significant spasmolytic effects in isolated rabbit jejunum and in ileal segments from rats and guinea pigs, modulating K^+^- and acetylcholine-induced, or spontaneous contractions [22,28,29]. In contrast, some other studies found rutin to be inactive in producing spasmolytic effects in gastrointestinal models [30,31,32].

Other *Ocimum* species were also investigated for their effects on gastrointestinal spasms. *O. selloi* essential oil attenuated guinea pig ileal contractility induced by carbachol, BaCl_2_, and low- and high-potassium solutions [33]. *O. gratissimum* essential oil induced relaxation of the spontaneous and abolished KCl- and acetylcholine-induced tonic contractions in guinea pig ileum [34,35]. While the primary constituents of the essential oil, eugenol and cineole, produced relaxation of KCl-induced contractility, their effect was weaker than that of the essential oil, implying that these compounds alone do not explain its relaxant activity [35].

## 4. Materials and Methods

### 4.1. Plant Material and Extraction Procedure

The plant material (*O. basilicum* leaves) was kindly provided by the food industry “Yumis” (Niš, Serbia). The material was supplied as dried basil leaf with an accompanying specification document confirming its identity as *O. basilicum,* cultivated in Egypt (the specification/batch number: S04-SP.BSI.018). According to the manufacturer’s specification (YUM-423-103.02, No. 34) the plant material used in this study was microbiologically safe and did not contain additives, preservatives, flavorings, or colorants. In addition, the maximum water content was 12%, the maximum total ash content 16%, acid-insoluble ash 2%, and the minimum content of volatile essential oil 0.3%. The plant material was free of impurities, foreign matter, live or dead insects, as well as insect parts or excreta. The material was stored in a dark and dry place until analysis.

The dried basil leaves were milled to a powder and used for the preparation of extracts. The prepared material was extracted with 96% *v*/*v*, 80% *v*/*v*, and 60% *v*/*v* ethanol (Reahem, Novi Sad, Serbia), absolute, 80% *v*/*v*, and 60% *v*/*v* methanol (T. T. T. d.o.o., Novaki, Croatia), at a ratio of 1:10, using ultrasound (sonication)-assisted extraction, digestion and maceration.

Ultrasound-assisted extraction was carried out by extracting the plant material in a thermostated ultrasonic bath Elmasonic S 40 H (220–240 V, 340 W, 37 Hz) (Elma Schmidbauer GmbH, Singen, Germany) for 40 min at 25 °C, after which the mixture was filtered and the solvent was completely removed using a rotary vacuum evaporator (IKA-Werke GmbH & Co. KG, Staufen, Germany). This procedure yielded extracts EU, E80U, E60U, MU, M80U, and M60U (solvents: 96% *v*/*v*, 80% *v*/*v*, and 60% *v*/*v* ethanol, and absolute, 80% *v*/*v* and 60% *v*/*v* methanol, respectively). Until analysis, the dry extracts were stored at 4 °C in a dark and cool place, in well-sealed glass containers.

Digestion, which involves extraction of powdered plant material with the solvent at elevated temperature, yielded extracts ED, E80D, E60D, MD, M80D, and M60D (solvents: 96% *v*/*v*, 80% *v*/*v*, and 60% *v*/*v* ethanol, and absolute, 80% *v*/*v* and 60% *v*/*v* methanol, respectively). The plant material was extracted for three hours at 40 °C, after which the liquid extract was evaporated to dryness using the rotary vacuum evaporator. Until analysis, the dry extracts were stored at 4 °C in a dark and cool place, in well-sealed glass containers.

Maceration was performed according to the Fourth Yugoslav Pharmacopoeia (Ph. Jug. IV) [36]. The extraction process lasted five days with shaking twice daily. On the final day, the mixture was filtered. After filtration, the macerates were allowed to stand for an additional two days in a cool and dark place, and were then evaporated to dryness using the rotary vacuum evaporator. In this manner, six extracts were obtained: EM, E80M, E60M, MM, M80M, and M60M (solvents: 96% *v*/*v*, 80% *v*/*v*, and 60% *v*/*v* ethanol, and absolute, 80% *v*/*v* and 60% *v*/*v* methanol, respectively). Until analysis, the dry extracts were stored at 4 °C in a dark and cool place, in well-sealed glass containers.

### 4.2. Chemical Characterization of the O. basilicum Leaf Extracts (HPLC-DAD Analysis)

Quantification of phenolic compounds was performed on an Agilent 1200 HPLC system (Agilent Technologies, Palo Alto, CA, USA) equipped with a diode array detector (DAD). Separation was achieved on a Purospher STAR RP-18e column (150 × 4.6 mm, particle size 5 μm; Merck, Darmstadt, Germany). Extracts were dissolved in HPLC-grade methanol (J.T. Baker, Deventer, The Netherlands) to a final concentration of 10 mg/mL and subsequently passed through a 0.45 μm membrane filter prior to injection. The injection volume was 10 μL, with a flow rate of 0.7 mL/min. The mobile phase consisted of 0.1% aqueous trifluoroacetic acid (Merck KGaA, Darmstadt, Germany) (solvent A) and acetonitrile (J.T. Baker, Deventer, The Netherlands) (solvent B), applied in a linear gradient program as follows: 0–3 min, 5% B; 3–32 min, 5–28% B; 32–44 min, 25–50% B; 44–52 min, 50–80% B; 52–54 min, 80–90% B; 54–59 min, 90–5% B; and 59–60 min, 5% B. The column temperature was maintained at 30 °C. Compound identification was based on comparison of retention times and UV-Vis spectra with those of authentic standards (rosmarinic acid, salvianolic acid B, cinnamic acid, caftaric acid, and chicoric acid—Sigma-Aldrich, St. Louis, MO, USA; chlorogenic acid and rutin—Acros Organics, Geel, Belgium; caffeic acid—Carl Roth GmbH + Co. Kg, Karlsruhe, Germany; salvigenin—Carbosynth Ltd., Compton, UK). Quantification was performed from peak areas using linear regression equations derived from calibration curves of the standards, and the results were expressed as milligrams per milligram of dry extract [37].

### 4.3. Spasmolytic Activity on Isolated Rat Ileum

#### 4.3.1. Experimental Animals

All experimental procedures were performed in accordance with the European Directive 2010/63/EU on the protection of animals used for scientific purposes and were approved by approved by the Animal Ethics Committee of the Faculty of Medicine, University of Niš (Decision No. 01-206-7). Male rats of the Wistar albino strain (200–250 g, 10–12 weeks old) were obtained from the vivarium of the Research Center for Biomedicine, Faculty of Medicine, University of Niš, Serbia. Animals were acclimatized for one week prior to the experiments and housed in stainless steel cages under standard laboratory conditions (room temperature 20–24 °C; 12 h light/dark cycle). Food and water were available ad libitum, except during the 24 h preceding the experiments, when food was restricted.

#### 4.3.2. Preparation of Rat Ileum

The rats were anesthetized by exposure to ether vapors. Following the induction of anesthesia, the thoracic cavity was opened and the aorta severed. Segments of the ileum, approximately 2 cm in length, were then excised and carefully separated from the mesentery. Each fragment was transferred into a 20 mL organ bath containing Tyrode’s solution, maintained at 37 °C, and continuously aerated with a gas mixture of 95% oxygen and 5% carbon dioxide. The composition of Tyrode’s solution was as follows: NaCl (150 mM), NaHCO_3_ (12 mM), glucose (5.5 mM), KCl (2.7 mM), MgCl_2_ (2 mM), CaCl_2_ (1.8 mM), and NaH_2_PO_4_ (0.4 mM) (all chemicals were manufactured by Centrohem, Stara Pazova, Serbia). Ileal segments were stretched and stabilized during a 30-min equilibration period prior to experimentation [37]. The extract solutions, which were added to the organ bath, were prepared by dissolving the extracts in distilled water with the aid of an ultrasonic bath. Changes in ileum contractility were recorded using a Transducer-TSZ-04-E system (Experimetria Ltd., Budapest, Hungary), and data acquisition and analysis were carried out with the SPEL Advanced ISOSYS software 1.0.

#### 4.3.3. Experimental Design

##### Effects of the *O. basilicum* Leaf Extracts on Spontaneous Ileum Contractility

In the first set of experiments, the influence of basil leaf extracts on spontaneous contractions of isolated rat ileum was examined. After an equilibration period, the extracts were administered in cumulative concentrations ranging from 0.005 to 1.5 mg/mL (0.005, 0.015, 0.05, 0.15, 0.5, and 1.5 mg/mL), enabling the construction of concentration–response curves. The spasmolytic effect at each concentration was expressed as the percentage of inhibition relative to the baseline spontaneous contractility of the ileum. Papaverine (Sigma-Aldrich, St. Louis, MO, USA), an alkaloid compound with well-documented spasmolytic activity, was used as a reference drug in concentrations of 0.01–3 μg/mL [37].

##### Effects of the *O. basilicum* Leaf Extracts on KCl-Induced Ileum Contractility

In the second series of experiments, the effects of basil leaf extracts on tonic ileum contractions induced by high potassium ion concentrations were investigated. Following an equilibration period, tonic contractions were elicited by adding KCl at a concentration of 80 mM, after which cumulative concentrations of the extracts (0.005, 0.015, 0.05, 0.15, 0.5, and 1.5 mg/mL) were applied at 15-min intervals. The relaxation of the KCl-precontracted ileum was expressed as a percentage of the control response induced by KCl. The same protocol was applied using verapamil (Sigma-Aldrich, St. Louis, MO, USA), a calcium channel blocker, in concentrations ranging from 0.015 to 1.5 μg/mL [37].

##### Effects of the *O. basilicum* Leaf Extracts on Acetylcholine-Induced Ileum Contractility

In the last series of experiments, the effects of basil leaf extracts were studied using acetylcholine-induced contractions of the ileum. A control dose–response curve was first generated based on contractility responses to cumulative concentrations of acetylcholine (5, 15, 50, 150, 500, and 1500 nM). The ileum segments were then rinsed with Tyrode’s solution until stable spontaneous contractions resumed. Each extract was subsequently added to the organ bath at concentrations of 0.5 mg/mL and 1.5 mg/mL, and after 5 min, the acetylcholine series was repeated. New dose–response curves were constructed to illustrate the effects of the extracts. The spasmolytic activity was expressed as the percentage of acetylcholine-induced contractility in the presence of the extracts compared to the control response. The same protocol was applied using atropine (Sigma-Aldrich, St. Louis, MO, USA) (140 nM), a non-selective muscarinic receptor antagonist, as a reference standard [37].

### 4.4. Statistical Analysis

Final results are presented as mean values of three (chemical characterization of the extracts) or six (spasmolytic effects of the extracts) parallel measurements with corresponding standard deviations. Statistical significance between or among mean values was evaluated using Student’s *t*-test or one-way ANOVA followed by Duncan’s post hoc test (*p* < 0.05 or *p* < 0.01). These statistical analyses were performed using SPSS version 20.0 (SPSS Inc., Chicago, IL, USA).

Principal component analysis (PCA) and hierarchical cluster analysis (HCA) were performed using MetaboAnalyst 6.0 software (https://www.metaboanalyst.ca/, accessed on 20 March 2026). Prior to analysis, raw data were autoscaled. Hierarchical clustering was conducted using Euclidean distance and Ward’s linkage method.

## 5. Conclusions

Basil (*O. basilicum*) has long been used in traditional medicine for the management of digestive disorders accompanied by abdominal pain. The present findings provide experimental evidence supporting the potential pharmacological basis of the ethnopharmacological use of basil, as basil leaf extracts obtained using different extraction procedures were shown to exert spasmolytic effects on ileal smooth muscle. Phytochemical analysis revealed that basil leaves represent a rich source of phenolic acids—including chlorogenic, caffeic, rosmarinic, salvianolic acid B, cinnamic, caftaric, and chicoric acids—as well as flavonoids such as salvigenin and rutin, which probably contribute to the observed spasmolytic effects. Among the identified constituents, rosmarinic acid was the predominant secondary metabolite across all samples, while rutin was the most abundant flavonoid. All tested extracts attenuated both spontaneous and pharmacologically induced contractions, with hydromethanolic extracts (M80M, M80U, and M60U) displaying the highest efficacy. Although the spasmolytic properties of *O. basilicum* have been previously reported, the relationship between the phytochemical composition of different basil extracts and their effects on intestinal smooth muscle contractility has remained insufficiently explored. The results of this study demonstrate that extracts of *O. basilicum* obtained using different solvents and extraction procedures differ in their phenolic composition and consequently in their spasmolytic activity. By evaluating these extracts in three experimental models of rat ileum contraction—spontaneous, KCl-induced, and acetylcholine-induced contractions—this study provides new insight into how variations in phenolic composition may influence different mechanisms of smooth muscle relaxation. Overall, the findings suggest that the spasmolytic activity of basil extracts is not determined by a single compound but rather by the combined contribution and possible synergistic interactions of multiple phenolic constituents present in the phytochemical matrix. In addition to the monitored phenolic compounds, future research should also consider other phytocompounds, including terpenes that are primarily components of the essential oil but may be present in minor amounts in the alcoholic and hydroalcoholic extracts, to determine how they contribute to the spasmolytic activity of basil extracts. Further studies are needed to clarify other mechanisms underlying the spasmolytic effects and to assess the therapeutic potential of basil leaf extracts in clinical settings.

## Figures and Tables

**Table 1:** Extraction yields (%) and contents of phenolic compounds (µg/mg dry extract) in the *Ocimum basilicum* L. leaf extracts obtained under different extraction conditions.

EXTRACT	Yields	Chlorogenic Acid	Caffeic Acid	Rosmarinic Acid	Salvianolic Acid B	Cinnamic Acid	Caftaric Acid	Chicoric Acid	Salvigenin	Rutin	Sum of All Compounds
	%	µg/mg									
EU	1.16	0.22 ± 0.00 ^a^	0.77 ± 0.02 ^a^	17.02 ± 0.08 ^a^	0.91 ± 0.05 ^a^	1.30 ± 0.01 ^a^	0.09 ± 0.00 ^a^	0.12 ± 0.00 ^a^	1.84 ± 0.09 ^a^	1.35 ± 0.04 ^a^	23.62
ED	2.70	0.52 ± 0.01 ^b^	2.25 ± 0.08 ^bc^	53.79 ± 0.04 ^b^	2.30 ± 0.01 ^b^	2.20 ± 0.02 ^b^	0.11 ± 0.00 ^a^	0.00 ± 0.00 ^a^	1.36 ± 0.08 ^b^	2.33 ± 0.06 ^b^	64.86
EM	3.93	0.58 ± 0.01 ^cd^	2.41 ± 0.09 ^d^	60.16 ± 0.06 ^c^	2.24 ± 0.02 ^b^	2.42 ± 0.04 ^c^	0.11 ± 0.00 ^a^	0.82 ± 0.01 ^b^	2.00 ± 0.07 ^c^	2.40 ± 0.02 ^b^	73.14
E80U	3.87	0.98 ± 0.02 ^e^	2.14 ± 0.02 ^b^	52.86 ± 0.07 ^d^	3.76 ± 0.04 ^c^	1.87 ± 0.08 ^d^	0.86 ± 0.02 ^b^	3.59 ± 0.09 ^c^	0.79 ± 0.01 ^d^	2.95 ± 0.01 ^c^	69.80
E80D	8.63	0.92 ± 0.03 ^fg^	2.50 ± 0.07 ^de^	61.88 ± 0.04 ^e^	7.31 ± 0.03 ^d^	1.22 ± 0.01 ^e^	1.45 ± 0.08 ^c^	6.94 ± 0.11 ^d^	0.75 ± 0.01 ^d^	4.52 ± 0.06 ^d^	87.49
E80M	8.83	0.95 ± 0.01 ^eg^	2.67 ± 0.06 ^f^	76.11 ± 1.06 ^f^	8.66 ± 0.06 ^e^	1.54 ± 0.05 ^f^	1.82 ± 0.01 ^d^	8.56 ± 0.12 ^e^	0.95 ± 0.02 ^e^	5.66 ± 0.05 ^e^	106.92
E60U	3.80	1.20 ± 0.06 ^h^	2.65 ± 0.04 ^fg^	63.73 ± 0.08 ^g^	6.07 ± 0.04 ^f^	1.83 ± 0.01 ^d^	0.98 ± 0.01 ^e^	5.02 ± 0.09 ^f^	0.73 ± 0.03 ^d^	4.01 ± 0.03 ^f^	86.22
E60D	9.27	0.83 ± 0.04 ^i^	2.55 ± 0.03 ^eg^	63.71 ± 0.07 ^g^	7.66 ± 0.04 ^g^	1.28 ± 0.05 ^ae^	1.68 ± 0.01 ^f^	7.52 ± 0.08 ^g^	0.75 ± 0.01 ^d^	4.63 ± 0.06 ^g^	90.61
E60M	8.80	0.89 ± 0.06 ^fjk^	2.54 ± 0.08 ^eg^	77.75 ± 0.91 ^h^	9.94 ± 0.10 ^h^	1.49 ± 0.03 ^f^	2.09 ± 0.02 ^g^	9.49 ± 0.12 ^h^	0.88 ± 0.02 ^f^	5.31 ± 0.07 ^h^	110.38
MU	2.90	0.00 ± 0.00 ^l^	1.70 ± 0.08 ^h^	56.03 ± 0.08 ^i^	4.54 ± 0.06 ^i^	2.77 ± 0.02 ^g^	0.49 ± 0.02 ^h^	2.78 ± 0.08 ^i^	2.30 ± 0.05 ^g^	3.17 ± 0.06 ^i^	73.78
MD	5.63	0.63 ± 0.01 ^d^	1.97 ± 0.07 ^i^	56.34 ± 0.12 ^i^	3.07 ± 0.04 ^i^	1.52 ± 0.01 ^f^	0.45 ± 0.01 ^h^	2.48 ± 0.09 ^j^	0.00 ± 0.00 ^h^	2.64 ± 0.03 ^j^	69.10
MM	6.87	0.53 ± 0.02 ^bc^	1.96 ± 0.06 ^i^	59.29 ± 0.26 ^j^	3.16 ± 0.01 ^j^	1.26 ± 0.01 ^ae^	0.44 ± 0.02 ^h^	2.27 ± 0.09 ^k^	1.19 ± 0.07 ^i^	1.27 ± 0.06 ^a^	71.37
M80U	7.23	0.90 ± 0.02 ^fgk^	1.90 ± 0.09 ^i^	62.14 ± 0.09 ^k^	7.19 ± 0.08 ^k^	1.08 ± 0.04 ^h^	1.39 ± 0.08 ^i^	5.41 ± 0.08 ^l^	0.39 ± 0.00 ^j^	3.09 ± 0.07 ^i^	83.49
M80D	10.77	0.88 ± 0.04 ^fijk^	2.24 ± 0.08 ^bc^	60.43 ± 0.05 ^c^	8.24 ± 0.09 ^l^	1.05 ± 0.06 ^h^	1.88 ± 0.01 ^d^	7.79 ± 0.06 ^m^	0.56 ± 0.00 ^k^	4.13 ± 0.07 ^k^	87.20
M80M	14.43	0.85 ± 0.02 ^ijk^	2.16 ± 0.01 ^b^	70.06 ± 0.10 ^e^	9.68 ± 0.09 ^m^	0.98 ± 0.01 ^i^	2.02 ± 0.03 ^j^	8.59 ± 0.14 ^e^	0.55 ± 0.00 ^k^	4.22 ± 0.08 ^l^	99.11
M60U	5.23	0.84 ± 0.03 ^ij^	1.95 ± 0.08 ^i^	49.97 ± 0.01 ^l^	3.56 ± 0.02 ^n^	1.37 ± 0.08 ^j^	0.88 ± 0.04 ^b^	3.50 ± 0.11 ^c^	0.54 ± 0.00 ^k^	2.71 ± 0.01 ^j^	65.32
M60D	6.87	0.91 ± 0.05 ^fg^	2.29 ± 0.10 ^c^	70.98 ± 0.11 ^m^	7.20 ± 0.06 ^k^	1.26 ± 0.04 ^ae^	1.06 ± 0.01 ^k^	0.00 ± 0.00 ^a^	0.58 ± 0.01 ^k^	3.94 ± 0.06 ^f^	88.22
M60M	12.90	0.36 ± 0.02 ^n^	1.73 ± 0.08 ^h^	49.26 ± 0.08 ^n^	6.70 ± 0.04 ^o^	0.00 ± 0.00 ^k^	1.56 ± 0.03 ^l^	7.03 ± 0.09 ^d^	0.00 ± 0.00 ^h^	2.66 ± 0.04 ^j^	69.30

EU, ED, EM—extracts prepared with 96% *v*/*v* ethanol by ultrasound (sonication)-assisted extraction, digestion and maceration, respectively; E80U, E80D, E80M—extracts prepared with 80% *v*/*v* ethanol by ultrasound-assisted extraction, digestion and maceration, respectively; E60U, E60D, E60M—extracts prepared with 60% *v*/*v* ethanol by ultrasound-assisted extraction, digestion and maceration, respectively; MU, MD, MM—extracts prepared with absolute methanol by ultrasound-assisted extraction, digestion, and maceration, respectively; M80U, M80D, M80M—extracts prepared with 80% *v*/*v* methanol by ultrasound-assisted extraction, digestion and maceration, respectively; M60U, M60D, M60M—extract prepared with 60% *v*/*v* methanol by ultrasound-assisted extraction, digestion, and maceration, respectively. The results represent the mean of the measurements ± standard deviations. Different superscript letters for each compound within the same column denote statistically significant differences among values based on ANOVA followed by Duncan’s post hoc test (*p* < 0.05).

**Table 2:** Inhibitory effects (%) of *Ocimum basilicum* L. extracts at the maximum tested concentration (1.5 mg/mL) on spontaneous rat ileum contractions.

EXTRACT	Max Inh.
	%
EU	7.18 ± 0.44 ^a^
ED	20.75 ± 1.61 ^b^
EM	15.25 ± 1.24 ^cd^
E80U	13.76 ± 0.98 ^d^
E80D	5.67 ± 0.11 ^a^
E80M	30.55 ± 1.69 ^e^
E60U	16.06 ± 1.27 ^cf^
E60D	16.93 ± 0.92 ^cf^
E60M	17.80 ± 0.88 ^f^
MU	32.72 ± 1.01 ^g^
MD	23.28 ± 1.54 ^h^
MM	44.48 ± 1.12 ^i^
M80U	29.20 ± 1.23 ^ek^
M80D	35.25 ± 1.11 ^j^
M80M	46.16 ± 2.11 ^i^
M60U	5.97 ± 0.88 ^a^
M60D	27.46 ± 0.95 ^k^
M60M	13.45 ± 0.94 ^d^
papaverine (0.003 mg/mL)	97.01 ± 0.98 ^l^

EU, ED, EM—extracts prepared with 96% *v*/*v* ethanol by ultrasound (sonication)-assisted extraction, digestion and maceration, respectively; E80U, E80D, E80M—extracts prepared with 80% *v*/*v* ethanol by ultrasound-assisted extraction, digestion and maceration, respectively; E60U, E60D, E60M—extracts prepared with 60% *v*/*v* ethanol by ultrasound-assisted extraction, digestion and maceration, respectively; MU, MD, MM—extracts prepared with absolute methanol by ultrasound-assisted extraction, digestion, and maceration, respectively; M80U, M80D, M80M—extracts prepared with 80% *v*/*v* methanol by ultrasound-assisted extraction, digestion and maceration, respectively; M60U, M60D, M60M—extract prepared with 60% *v*/*v* methanol by ultrasound-assisted extraction, digestion, and maceration, respectively. The results represent the mean of the measurements ± standard deviations. Different superscript letters for each compound within the same column denote statistically significant differences among values based on ANOVA followed by Duncan’s post hoc test (*p* < 0.05).

**Table 3:** Response (%) at the maximum tested concentration (1.5 mg/mL) of *Ocimum basilicum* L. extracts in KCl-induced contractions of rat ileum.

EXTRACT	Max Response
	%
EU	68.18 ± 3.32 ^abc^
ED	71.83 ± 4.44 ^a^
EM	69.57 ± 1.56 ^ab^
E80U	64.22 ± 1.55 ^cde^
E80D	64.72 ± 2.45 ^cd^
E80M	61.82 ± 3.43 ^de^
E60U	72.56 ± 2.62 ^a^
E60D	66.15 ± 1.43 ^bcd^
E60M	59.74 ± 2.23 ^e^
MU	61.55 ± 1.66 ^de^
MD	64.40 ± 0.55 ^cde^
MM	68.34 ± 2.38 ^abc^
M80U	59.48 ± 3.34 ^e^
M80D	67.37 ± 2.46 ^bcd^
M80M	62.15 ± 1.51 ^de^
M60U	64.03 ± 0.44 ^cde^
M60D	65.62 ± 1.39 ^bcd^
M60M	63.90 ± 1.34 ^cde^
Verapamil (0.0015 mg/mL)	5.04 ± 0.09 ^f^

EU, ED, EM—extracts prepared with 96% *v*/*v* ethanol by ultrasound (sonication)-assisted extraction, digestion and maceration, respectively; E80U, E80D, E80M—extracts prepared with 80% *v*/*v* ethanol by ultrasound-assisted extraction, digestion and maceration, respectively; E60U, E60D, E60M—extracts prepared with 60% *v*/*v* ethanol by ultrasound-assisted extraction, digestion and maceration, respectively; MU, MD, MM—extracts prepared with absolute methanol by ultrasound-assisted extraction, digestion, and maceration, respectively; M80U, M80D, M80M—extracts prepared with 80% *v*/*v* methanol by ultrasound-assisted extraction, digestion and maceration, respectively; M60U, M60D, M60M—extract prepared with 60% *v*/*v* methanol by ultrasound-assisted extraction, digestion, and maceration, respectively. The results represent the mean of the measurements ± standard deviations. Different superscript letters for each compound within the same column denote statistically significant differences among values based on ANOVA followed by Duncan’s post hoc test (*p* < 0.05).

**Table 4:** Response (%) of *Ocimum basilicum* L. extracts at concentrations of 0.5 mg/mL and 1.5 mg/mL on maximal (100%) acetylcholine-induced contractions of rat ileum.

EXTRACT	Max Response Ach	Max Response Ach + 0.5 mg/mL	Max Response Ach + 1.5 mg/mL
	%		
EU	100 ^a^	75.98 ± 0.75 ^abcd^	66.41 ± 1.21 ^ab^
ED	100 ^a^	85.71 ± 1.50 ^efgh^	67.92 ± 1.56 ^ab^
EM	100 ^a^	83.29 ± 6.15 ^efgi^	76.69 ± 2.77 ^c^
E80U	100 ^a^	85.21 ± 1.56 ^efgh^	69.47 ± 0.85 ^a^
E80D	100 ^a^	78.43 ± 0.71 ^bcdi^	67.11 ± 2.29 ^ab^
E80M	100 ^a^	88.25 ± 3.99 ^fh^	69.61 ± 0.96 ^a^
E60U	100 ^a^	86.71 ± 4.36 ^fgh^	67.04 ± 3.95 ^ab^
E60D	100 ^a^	80.07 ± 3.74 ^cdei^	63.48 ± 1.41 ^bd^
E60M	100 ^a^	73.42 ± 2.82 ^abj^	59.92 ± 2.31 ^def^
MU	100 ^a^	74.66 ± 4.91 ^abcj^	52.26 ± 4.74 ^g^
MD	100 ^a^	81.20 ± 3.51 ^degi^	55.90 ± 2.81 ^gh^
MM	100 ^a^	70.59 ± 3.41 ^aj^	48.36 ± 1.37 ^i^
M80U	100 ^a^	69.25 ± 1.84 ^j^	45.76 ± 1.34 ^i^
M80D	100 ^a^	70.42 ± 4.54 ^aj^	58.22 ± 1.75 ^fh^
M80M	100 ^a^	50.71 ± 1.36 ^k^	39.95 ± 2.28 ^j^
M60U	100 ^a^	68.83 ± 4.98 ^j^	35.74 ± 1.15 ^k^
M60D	100 ^a^	94.61 ± 2.68 ^l^	62.35 ± 1.83 ^de^
M60M	100 ^a^	90.56 ± 3.23 ^hl^	58.80 ± 2.49 ^efh^
atropine	100 ^a^	16.02 ± 0.68 ^m^ (Ach + 140 nM)	

EU, ED, EM—extracts prepared with 96% *v*/*v* ethanol by ultrasound (sonication)-assisted extraction, digestion and maceration, respectively; E80U, E80D, E80M—extracts prepared with 80% *v*/*v* ethanol by ultrasound-assisted extraction, digestion and maceration, respectively; E60U, E60D, E60M—extracts prepared with 60% *v*/*v* ethanol by ultrasound-assisted extraction, digestion and maceration, respectively; MU, MD, MM—extracts prepared with absolute methanol by ultrasound-assisted extraction, digestion, and maceration, respectively; M80U, M80D, M80M—extracts prepared with 80% *v*/*v* methanol by ultrasound-assisted extraction, digestion and maceration, respectively; M60U, M60D, M60M—extract prepared with 60% *v*/*v* methanol by ultrasound-assisted extraction, digestion, and maceration, respectively. The results represent the mean of the measurements ± standard deviations. Different superscript letters for each compound within the same column denote statistically significant differences among values based on ANOVA followed by Duncan’s post hoc test (*p* < 0.05).

## Data Availability

The raw data is available upon request from the authors.

## Supplementary Materials

The following supporting information can be downloaded at: https://www.mdpi.com/article/10.3390/plants15071079/s1, Figure S1: Inhibitory effects of atropine (A) and the most active *Ocimum basilicum* L. extracts, M60U (B) and M80M (C), on acetylcholine-induced contractions of rat ileum. Each point represents the mean value of percentages of maximal response ± SD of six segments (Student’s *t*-test, * *p* < 0.05, and ** *p* < 0.01 vs. control).
