# Supplementary material for: Effects of Basil (Ocimum basilicum L.) Leaf Extracts on Gastrointestinal Smooth Muscle Spasms: An In Vitro Study on Rat Ileum
Source: Plants (Basel). 2026 Apr 1;15(7):1079. doi: 10.3390/plants15071079 (PMC13074676; doi:10.3390/plants15071079)
Supplement: Supplementary file 1 [file plants-15-01079-s001.zip › plants-4190408-supplementary.pdf]

Supplementary file

Effects of Basil (*Ocimum basilicum* L.) Leaf Extracts on Gastrointestinal Smooth Muscle Spasms: An In Vitro Study on Rat Ileum

Milica Randjelović[1]*, Nebojša Simić[2]*, Suzana Branković[3], Maja Koraćević[4], Miloš Jovanović[1], Nemanja Kitić[3], Bojana Miladinović[1], Milica Milutinović[1], Dušanka Kitić[1]

[1] Department of Pharmacy, Faculty of Medicine, University of Nis, Ave. Dr. Zorana Djindjica 81, 18000 Nis, Serbia; milos.jovanovic@medfak.ni.ac.rs (M.J.); bojana.miladinovic@medfak.ni.ac.rs (B.M.); milica.milutinovic@medfak.ni.ac.rs (M.M.); dusanka.kitic@medfak.ni.ac.rs (D.K.)

[2] Department of Chemistry, Norwegian University of Science and Technology, 7491 Trondheim, Norway

[3] Department of Physiology, Faculty of Medicine, University of Nis, Ave. Dr. Zorana Djindjica 81, 18000 Nis, Serbia; suzana.brankovic@medfak.ni.ac.rs (S.B.); nemanja.kitic@medfak.ni.ac.rs (N.K.)

[4] Faculty of Medicine, University of Nis, Ave. Dr. Zorana Djindjica 81, 18000 Nis, Serbia; koracevic.maja@gmail.com (M.K.)

* Correspondence: milica.randjelovic@medfak.ni.ac.rs (M.R.); nebojsa.simic@ntnu.no (N.S.)

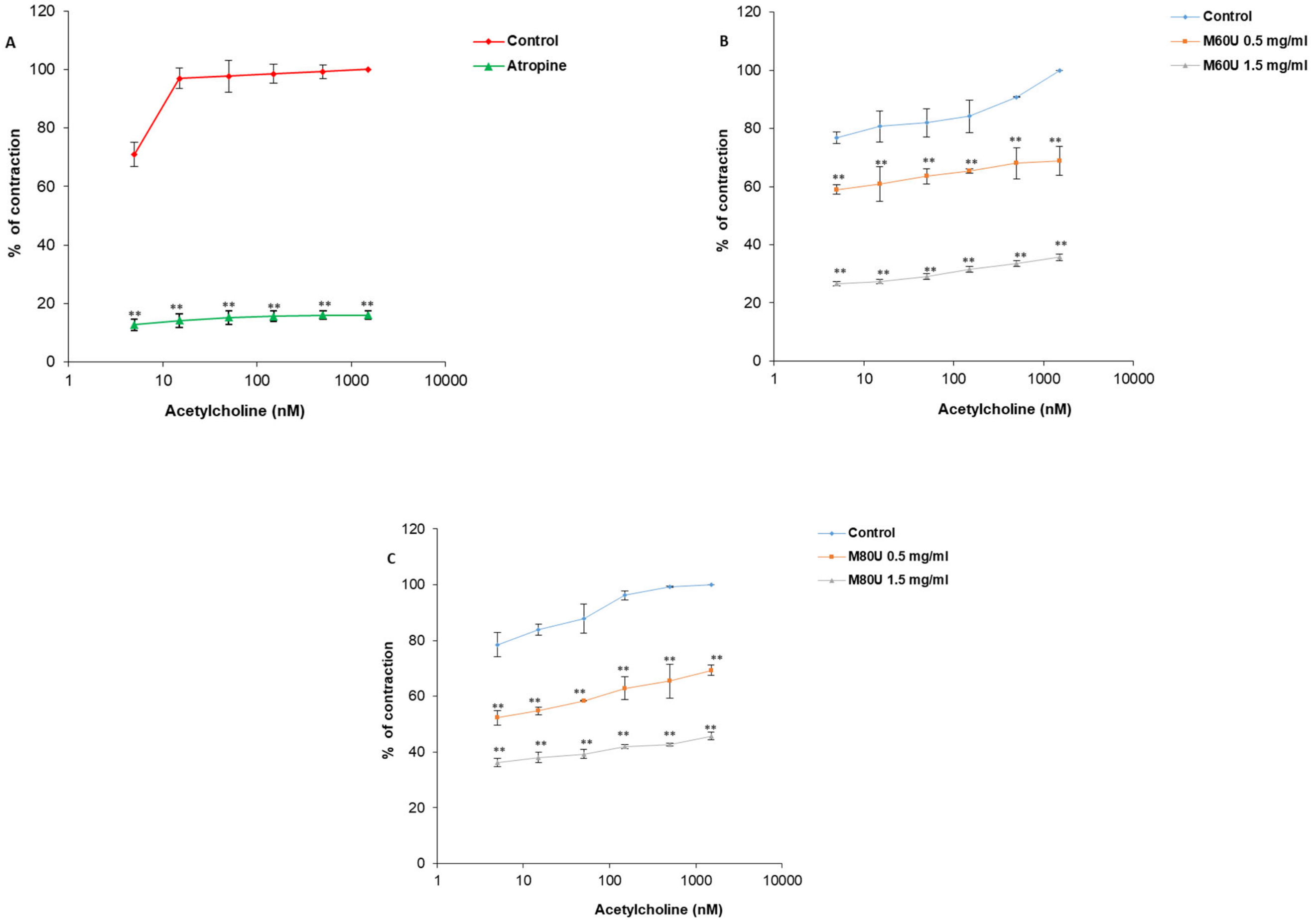

Figure S1: Inhibitory effects of atropine (**A**) and the most active *Ocimum basilicum* L. extracts, M60U (**B**) and M80U (**C**), on acetylcholine-induced contractions of rat ileum. Each point represents the mean value of percentages of maximal response ± SD of six segments (Student's *t*-test, * $p < 0.05$, and ** $p < 0.01$ vs. control).
